# Shortening identification times: comparative observational study of three early blood culture testing protocols

**DOI:** 10.3389/fcimb.2023.1192002

**Published:** 2023-07-18

**Authors:** Paul-Antoine Chatelard, Nathalie Rousseau, Laurence Parmeland, Pierre Metral, Caroline Pariset, Emmanuel Vivier

**Affiliations:** ^1^ Centre Hospitalier Saint Joseph Saint Luc, Médecine Intensive Réanimation, Lyon, France; ^2^ Centre Hospitalier Saint Joseph Saint Luc, Laboratoire de biologie médicale, Lyon, France; ^3^ Centre Hospitalier Saint Joseph Saint Luc, Département d’Information Médicale, Lyon, France; ^4^ Centre Hospitalier Saint Joseph Saint Luc, Maladies Infectieuses, Lyon, France; ^5^ Centre Hospitalier Saint Joseph Saint Luc, Unité de Recherche Clinique, Lyon, France

**Keywords:** bacteremia, MRSA/SA PCR, ePlex assay, *β*-Lacta test, oxidase test

## Abstract

**Background:**

While early appropriate antibiotic therapy is a proven means of limiting the progression of infections, especially bacteremia, empirical antibiotic therapy in sepsis is ineffective up to 30%. The aim of this study was to compare early blood culture testing protocols in terms of their ability to shorten the delay between blood sampling and appropriate antibiotic therapy.

**Methods:**

In this french observational study, we compared three blood culture testing protocols. Positive blood cultures were tested using either GenMark ePlex panels (multiplex PCR period), a combination of MRSA/SA PCR, *β*-Lacta and oxidase tests (multitest period), or conventional identification and susceptibility tests only (reference period). Conventional identification and susceptibility tests were performed in parallel for all samples, as the gold standard.

**Results:**

Among the 270 patients with positive blood cultures included, early and conventional results were in good agreement, especially for the multitest period. The delay between a blood culture positivity and initial results was 3.8 (2.9–6.9) h in the multiplex PCR period, 2.6 (1.3–4.5) h in the multitest period and 3.7 (1.8–8.2) h in the reference period (p<0.01). Antibiotic therapy was initiated or adjusted in 68 patients based on early analysis results. The proportion of patients receiving appropriate antibiotic therapy within 48 h of blood sampling was higher in the multiplex PCR and multitest periods, (respectively 90% and 88%) than in the reference period (71%).

**Conclusion:**

These results suggest rapid bacterial identification and antibiotic resistance tests are feasible, efficient and can expedite appropriate antibiotic therapy.

## Background

Bloodstream infections are associated with high morbidity and mortality and the increase in multidrug resistant pathogens has made them increasingly difficult to treat (Rhodes et al., 2017; Robineau et al., 2018; Cassini et al., 2019). Identifying causative pathogens is crucial to optimize treatment and patient outcomes (Seifert, 2009); however, conventional identification and antimicrobial susceptibility tests are time-consuming, with results only available 48–72 h after blood culture collection (Miller et al., 2018; CASFM/EUCAST, 2019). Although empirical antibiotic therapy can be adjusted based on the Gram stain and bacterial species results 24–48 h after bacterial growth positivity, a further 24–48 h is required for definitive susceptibility results to confirm or correct the antibiotic therapy. This delay between blood sampling and appropriate antibiotic therapy is a cornerstone in the management of patients with sepsis and should be reduced as much as possible (Rivers et al., 2001; Rhodes et al., 2017). Up to 20 or 30% of patients with sepsis are initially treated with inappropriate empirical antibiotic therapy (Yokota et al., 2014), and delayed or inappropriate antibiotic therapy is strongly associated with mortality (Kollef, 2000; Leone et al., 2003; Kumar, 2010; Rhodes et al., 2017; Robineau et al., 2018). New, potentially more efficient techniques based on multiplex PCR (m-PCR), targeted gene sequencing or enzymatic activity testing are particularly interesting in this context.

In this study, we compared in three consecutive periods, three blood culture testing strategies based on m-PCR and multiple rapid tests with conventional Gram staining, to investigate whether the new approaches are feasible and shorten the time to appropriate antibiotic therapy. The main objective of the study was to determine the feasibility and performance of multiple rapid tests and m-PCR tests in clinical practice and compare the effectiveness of the two methods. The secondary objective was to determine whether either approach reduced the delay between blood sampling and appropriate antibiotic therapy.

## Materials and methods

### Study design

**Figure 1:**
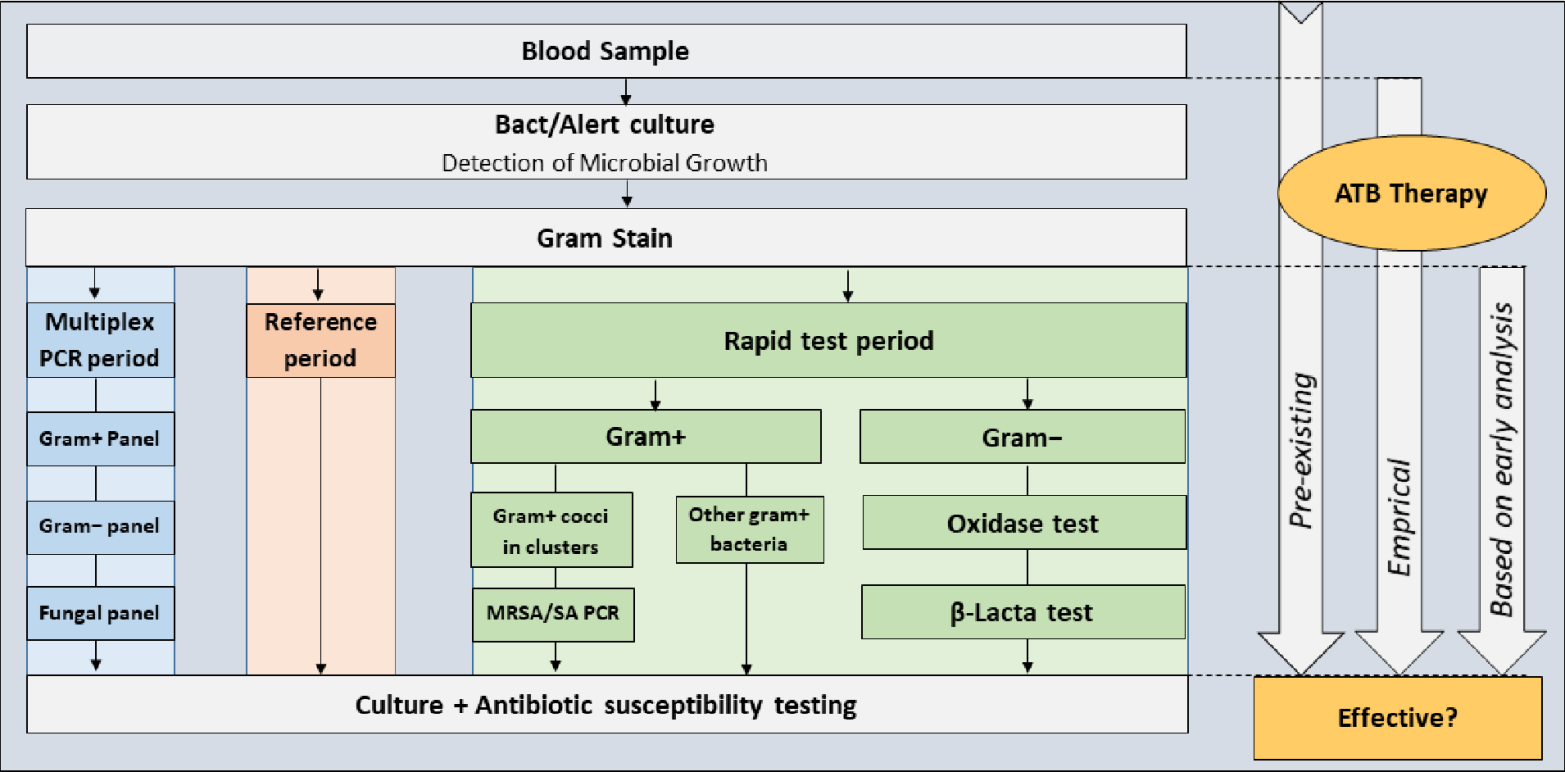
Testing flow diagram.

This observational study included all patients older than 18 years with positive blood cultures treated in two non-university hospitals in Lyon, France between 1 March and 20 September 2019. Between 1 March and 30 April 2019 (the m-PCR period), blood cultures were tested using GenMark ePlex blood culture identification panels, between 30 April and 21 July 2019 (the reference period), blood cultures were tested by conventional methods, and between 22 July and 20 September 2019 (the multitest period), a combination of rapid MRSA/SA PCR, *β*-Lacta and oxidase tests (**Figure 1**). All early analysis (m-PCR, rapid tests and Gram stain), were performed 24 hours a day and 7 days a week. The same number of cultures were analyzed in each period. For safety reasons and to evaluate the performance of the rapid techniques, conventional identification and susceptibility tests were performed in parallel on all samples as the gold standard. All tests were performed in the same laboratory (Saint-Joseph-Saint-Luc Hospital, Lyon, France). Additional details are provided in the **Supplemental Material**.

### Laboratory procedures

All blood samples were immediately incubated on a round-the-clock basis in a BACT/ALERT 3D instrument (BioMérieux, France*)* and Gram staining was immediately performed on positive cultures. Bacterial identification and antimicrobial susceptibility tests were then performed on positive blood cultures by microbiology laboratory staff during working hours (Mon.–Fri., 8 a.m. to 7 p.m.; Sat., 8 a.m. to 1 p.m.) using a VITEK 2 system (BioMérieux, France*)*.

During the m-PCR period, blood cultures were tested using the ePlex blood culture identification panel system (GenMark Diagnostics, Carlsbad, CA, USA) (Schmitz and Tang, 2018). The ePlex blood culture identification panel (EU CE-IVD certification in 2017) consists of three separate cartridges for gram positive, gram negative, and fungal pathogens, and several genus- and/or species-level probes. The gram-positive and gram-negative cartridges also include several probes of key antimicrobial resistance genes (**Table 1**).

**Table 1:** Microorganisms and resistance genes detected by the different cartridges in the ePlex blood culture identification panel.

Identification	GRAM positive Panel	GRAM negative Panel	FONGIC Panel
***Microorganisms***	*Bacillus cereus* group *Bacillus subtilis* group *Corynebacterium* Cutibacterium acnes (P. acnes) Enterococcus Enterococcus faecalis Enterococcus faecium Lactobacillus Listeria Listeria monocytogenes Micrococcus Staphylococcus Staphylococcus aureus Staphylococcus epidermidis Staphylococcus lugdunensis Streptococcus Streptococcus agalactiae Streptococcus anginosus group Streptococcus pneumoniae Streptococcus pyogenes Pan Gram-negative Pan Candida	*Acinetobacter baumannii* *Bacteroides fragilis* *Citrobacter* *Cronobacter sakazakii* *Enterobacter (non-cloacae* complex) *Enterobacter cloacae* complex *Escherichia coli* *Fusobacterium nucleatum* *Fusobacterium necrophorum* *Haemophilus influenzae* *Klebsiella oxytoca* *Klebsiella pneumoniae* group *Morganella morganii* *Neisseria meningitidis* Proteus *Proteus mirabilis* Pseudomonas aeruginosa Salmonella Serratia Serratia marcescens Stenotrophomonas maltophilia Pan Gram-Positive Pan Candida	*Candida albicans* *Candida dubliniensis* *Candida famata* *Candida glabrata* *Candida guilliermondii* *Candida kefyr* *Candida lusitaniae* *Candida krusei* *Candida parapsilosis* *Candida tropicalis* *Cryptococcus neoformans* *Cryptococcus gattii* *Fusarium* *Malassezia furfur* *Rhodotorula* *Trichosporon*
*Resistance genes*	*MecA* *MecC* *VanA* *VanB*	*CTX-M* *IMP* *KPC* *NDM* *OXA-23* *OXA-48* *VIM*	

During the multiple rapid test (multitest) period, blood cultures were tested based on Gram stain results using a combination of three rapid tests with a decision algorithm to select the best possible combination based on Gram-staining results (**Figure 1**): Bactident oxidase tests (Merck, Darmstadt, Germany) *β*-Lacta tests (Bio-Rad, Marnes la Coquette, France) for gram negative bacteria, and MRSA/SA tests for clustered gram positive cocci (GeneXpert MRSA/SA test, Cepheid, Sunnyvale, CA).

During the reference period, the gram stain was considered as the “early analysis” and was communicated to the physicians to help them to adapt empirical antibiotic therapy. The term was also changed in **Table 2**.

**Table 2:** Patient characteristics and microbiological results.

	m-PCR period (n= 90)	Reference period (n= 90)	Multitest period (n= 90)	*p*
Age (years)	70 ± 20	70 ± 16	71 ± 20	0.487
< 65 years	26 (29)	30 (33)	25 (28)	0.690
65–80 years	30 (33)	35 (39)	25 (28)	0.287
≥ 80 years	34 (38)	25 (28)	40 (44)	0.065
Male	47 (52)	51 (57)	45 (50)	0.659
Contamination	9 (10)	8 (9)	7 (8)	0.872
Nosocomial infection	22 (24)	33 (37)	31 (34)	0.172
Pneumonia	22 (24)	12 (13)	21 (23)	0.125
Urinary tract infection	16 (18)	28 (31)	22 (24)	0.115
Septic shock	16 (18)	9 (10)	16 (18)	0.244
ICU admission	28 (31)	20 (22)	26 (29)	0.380
SAPS II score on admission	40 ± 13	38 ± 15	52 ± 22	0.040
Catecholamines	16 (18)	9 (10)	19 (21)	0.117
Invasive ventilation	16 (18)	8 (9)	14 (16)	0.203
Renal replacement therapy	7 (8)	4 (4)	8 (9)	0.479
In-hospital deaths	12 (13)	6 (7)	13 (14)	0.209
Length of hospital stay (days)	9 (4–23)	9 (4–18)	13 (4–25)	0.212
All pathogens identified	90 (100)	90 (100)	84 (93)	0.417
Gram-positive	45 (45)	44 (44)	51 (55)	0.410
Gram-negative	54 (54)	55 (55)	40 (43)	0.193
Yeasts	1 (1)	1 (1)	2 (2)	0.776
Time from blood sampling to positive blood culture (hours)	16.9 (13–22.1)	13.7 (11.9–21.5)	15.9 (13–21.3)	0.057
Time from positive blood culture to early analysis results (hours)	3.8 (2.9–6.9)	3.7 (1.8–8.2)	2.6 (1.3–4.5)	<0.01
Time from positive blood culture to final results (hours)	51.8 (43.8–66.5)	49.3 (37.5–58)	48.9 (40.1–54.6)	0.181
Time from blood sampling to final results (hours)	68.9 (61.2–93.4)	62.8 (56.9–74.3)	64.5 (58.7–75)	0.031

Data are reported as mean ± standard deviation, median (interquartile range) or frequency (%).

### Clinical guidelines

All blood culture results were immediately communicated to the attending physician. Treatment protocols and guidelines were established for each period by a working group of clinical biochemists, infectious diseases specialists, and intensive care physicians (**Supplemental Table 2**). The protocols were accessible on the computer system of the two hospitals. These measures were implemented alongside the rapid techniques to optimize their impact on patient outcomes (Banerjee et al., 2015; Vardakas et al., 2015; Barlam et al., 2016; Timbrook et al., 2017; De Waele et al., 2018).

### Data collection and outcomes

The following data were collected from the patients’ electronic medical records: age, gender, identified pathogens, infection sites, ICU admission, SAPS 2 score, need for vasopressor therapy, invasive mechanical ventilation or renal replacement therapy, length of hospital stay and mortality. All microbiological results were collected. The final results of the conventional identification and susceptibility tests were used as reference to assess the results of the m-PCR and rapid tests. The times of blood sampling, positive blood culture alerts, early analysis results and final results were recorded. The timing and choice of antibiotics were recorded and defined for each step: pre-existing antibiotic therapy (started before blood sampling), empirical antibiotic therapy (initiated between the sample collection and antibiogram result) and antibiotic therapy based on early analysis results (started and selected based on early analysis results and antibiotic treatment guidelines, see **Supplemental Tables 1** and **2**). Antibiotic therapy wad considered effective according to the definitive result of the antibiogram.

The primary outcome measures were the feasibility of the methods and the diagnostic performance of the rapid tests relative to conventional tests (sensitivity, specificity, positive and negative predictive values). We also evaluated the time between blood culture positivity and test results for each approach.

The clinical impact of each technique was assessed in terms of the time from blood culture collection to the introduction of appropriate antibiotic therapy. These delays were compared between patients treated in the different periods and for subgroups of patients with confirmed bacteremia and with or without appropriate antibiotic therapy at the release of early analysis results. Patient outcomes were evaluated in terms of ICU admission, length of hospital stay and in-hospital mortality. Duration of exposure to broad-spectrum antibiotics was also considered.

### Statistical analysis

Continuous variables were expressed as median (interquartile range), and categorical variables were expressed as numbers and percentages. Between-group comparisons were performed using ANOVA tests (if the data were normally distributed) or Kruskal-Wallis tests (if the distribution was skewed) for continuous variables and using chi-square tests for categorical variables. A p-value <0.05 was deemed significant. All analyses were performed with the software SPSS (version 20.0, SPS Inc, Chicago, Illinois). The diagnostic performance of the rapid tests (sensitivity and specificity) were calculated using the results of the conventional tests as the gold standard (see **Supplemental Material**).

## Results

### Population and pathogens

Two hundred and seventy patients were included in total, 90 in each of the three periods, from the medical, geriatric, surgical, emergency, and intensive care units. All patients had a positive blood culture with an unknown pathogenic microorganism in the preceding 48 hours. Patient characteristics and biochemical results are summarized in **Table 2**. Patients treated in the multitest period had higher SAPS 2 scores. The bloodstream infections were mostly due to a single pathogen but 21/270 (8%) involved multiple pathogens. The main sources of the infection were the urinary tract (24% of cases) and the lungs (19%), and the most common pathogens were *Escherichia coli* and *Staphylococcus aureus* (found in 24% and 10% of blood cultures, respectively). The gram distribution was similar in the three periods (51% of gram-negative bacteria and 47% of gram-positive bacteria). A small proportion of cases (1% in each period) involved fungal infections (**Table 2**). The median time between blood sampling and blood culture positivity tend to be shorter in the reference period than in the other two periods (13.7 h vs 15.9 and 16.9 h, *p* = 0.06)

### Feasibility and performance of the rapid tests

The performance of the m-PCR tests was variable with sensitivities for bacterial identification and antibiotic resistance of 93% and 78%, respectively, specificities of 40% and 100%, positive predictive values of 89% and 100%, and negative predictive values of 55% and 98%, respectively (see **Supplemental Material 3**). The performance of the multiple rapid tests was excellent with sensitivities, specificities, and negative and positive predictive values of 100% for bacterial identification and susceptibility results. The multitest method was also the fastest with delays between blood sampling and early analysis results of 3.8 (2.9–6.9) h, 3.7 (1.8–8.2) h and 2.6 (1.3–4.5) h in the m-PCR, reference and multitest periods, respectively (p < 0,01; **Table 2**).

### Adjustment of antibiotic therapy based on rapid test results

**Figure 2:**
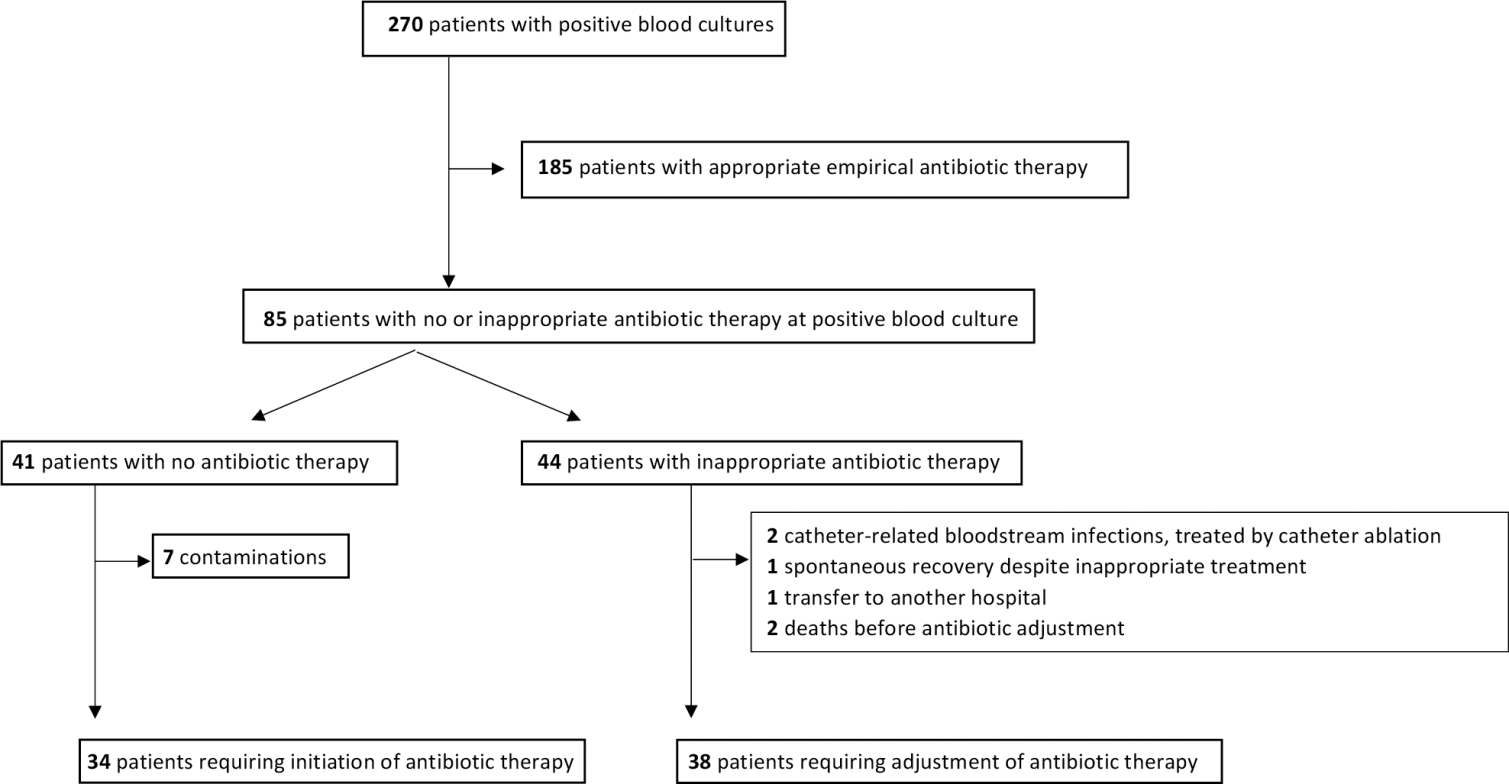
Treatment flow diagram.

Empirical antibiotic therapy was initiated in 229 patients (85%) before early analysis results were available and was inappropriate in 44 of these patients (19%) before early analysis results (**Figure 2**). For these patients, the delay between blood sampling and analysis results. For these patients, the time from blood sampling to the initiation of appropriate antibiotic therapy did not differ between the three periods. Antibiotic therapy was initiated or adjusted based on early analysis results in 78 patients in total (introduced in 34 patients without prior antibiotic therapy and adjusted in 44 patients with inappropriate empirical antibiotic therapy). Among these 78 patients, the delay between blood sampling and appropriate antibiotic therapy was 9.5 h (29%) shorter in the m-PCR period and 8.9 h (27%) shorter in the multitest period than in the reference period. The use of m-PCR or multiple rapid tests was associated with a higher likelihood of patients receiving appropriate antibiotic therapy within 24 and 48 h of blood sampling. Among patients with no or inappropriate antibiotic therapy prior to early analysis results, the proportions receiving appropriate antibiotic therapy in the m-PCR, rapid test and reference periods increased to 60%, 50% and 42%, respectively, within 24 h of blood sampling, and 90%, 88% and 71% within 48 h of blood sampling. (**Table 3**).

**Table 3:** Treatment implications and delays (contaminations excluded).

	m-PCR period	Reference period	Multitest period	*p*
All patients (n=246)				
Time (hours) from blood sampling to appropriate antibiotic therapy	2.8 (0.5–17.5)	6 (1.6–20.3)	6.4 (1–18.7)	0.196
Appropriate antibiotic therapy < 24 h after blood sampling	69/81 (85)	62/82 (76)	66/83 (80)	0.421
Appropriate antibiotic therapy < 48 h after blood sampling	77/81 (95)	69/82 (84)	76/83 (92)	0.105
**No AT before rapid test results (n=34)**	10/81 (12)	12/82 (15)	12/83 (14)	0.972
Time (hours) from blood sampling to appropriate antibiotic therapy (n=30)*	22.1 (18.1–24.9)	25 (20.3–33.1)	23.4 (15.9–24.6)	0.416
**Empirical antibiotic therapy before early analysis (n=212)**	71/81 (88)	70/82 (85)	71/83 (86)	0.972
**Inappropriate antibiotic therapy before early analysis (n=44)**	12/71 (17)	17/70 (24)	15/71 (21)	0.555
Time (hours) from blood sampling to appropriate antibiotic therapy (n=38)**	28.5 (20.7–36.5)	47.6 (22.4–58.1)	24 (18.2–35.3)	0.264
AT introduction or adjustment after early analysis (n=68)				
Time (hours) from blood sampling to appropriate antibiotic therapy (hours)	23.4 (19.9–29.7)	32.9 (20.3–51.5)	24 (17.7–30.8)	0.454
Appropriate antibiotic therapy < 24 h after blood sampling	12/20 (60%)	10/24 (42%)	12/24 (50%)	0.480
Appropriate antibiotic therapy < 48 h after blood sampling	18/20 (90%)	17/24 (71%)	21/24 (88%)	0.180

Data are reported as mean ± standard deviation, median (interquartile range) or frequency (%).

*4/34 patients never received antibiotic therapy (one transient bacteriemia in the reference period, one transfer to another hospital before antibiotic therapy was initiated in the reference period, and palliative care for two patients in the multitest period).

**6/44 patients never received antibiotic therapy (two deaths in the m-PCR and reference periods, one lung infection resolved under inappropriate antibiotic therapy in the m-PCR period, two catheter infections treated by catheter ablation in the reference and multitest periods, and one transfer to another hospital before appropriate antibiotic therapy in the reference period.

### Patient outcomes and antibiotic consumption

The in-hospital mortality rate was 11.5% overall (31/270) and was slightly lower (but not statistically significant) in the reference period (7%) than in the m-PCR and multitest periods (13% and 14%, respectively, p = 0.209). The median length of hospital stay was 9 (4–22.5) days and did not differ between periods. Broad-spectrum antibiotic consumption was similar in the three periods (**Supplemental Table 4**).

## Discussion

### Rapid techniques

Faster identification of bacterial species and antibiotic resistance could allow earlier administration of appropriate narrow-spectrum antibiotics and should thereby help improve patient outcomes, reduce costs, adverse effects, and the emergence of antibiotic resistant organisms (Caliendo et al., 2013; Garnier et al., 2017). This has prompted manufacturers to develop m-PCR systems designed to rapidly identify causative organisms in sepsis and common antibiotic resistance genes. Molecular diagnostic assays are now available that can be used directly on positive blood culture bottles, providing results much faster than conventional cultures and antimicrobial susceptibility testing (Liesenfeld et al., 2014; Salimnia et al., 2016; Huang et al., 2019; Oberhettinger et al., 2020). The good diagnostic performance of m-PCR is well-established (Banerjee et al., 2015; Southern et al., 2015; Walker et al., 2016; Schmitz and Tang, 2018; Bryant et al., 2020; Carroll et al., 2020; Krifors et al., 2020), but just like for other rapid techniques, few studies have shown any significant clinical impact (Timbrook et al., 2017; Rodrigues et al., 2019). The ePlex blood culture identification panel is a hybrid m-PCR system that identifies a panel of genes from pathogenic organisms or associated with antibiotic resistance. This panel has been shown to identify around 95% of frequently encountered pathogens with a sensitivity and specificity of more than 90% (Huang et al., 2019; Bryant et al., 2020; Carroll et al., 2020; Krifors et al., 2020; Oberhettinger et al., 2020). The present results confirm the rapid nature of the test but suggest that its efficacy may be lower than previously reported (particularly for antibiotic resistance findings).

The other rapid testing protocol investigated in this study involved multiple rapid tests (Parmeland et al., 2021) with a decision algorithm to select the best possible combination based on Gram-staining results. The GeneXpert MRSA/SA PCR test is a genotypic test able to detect methicillin-resistant *Staphylococcus* with a sensitivity and specificity close to 100% in blood cultures (Parta et al., 2009; Brown and Paladino, 2010; Davies et al., 2012). The *β*-Lacta test is a phenotypic test that detects *β*-lactamase–producing enterobacteria with third generation-cephalosporin resistance with a reported sensitivity around 85% and a specificity of more than 95% in previous studies (Renvoise et al., 2013; Compain et al., 2015; Garnier et al., 2017; Hasso et al., 2017). The oxidase test is also a phenotypic test used to detect gram-negative bacteria producing cytochrome oxidase, typically *Pseudomonas* species in bacteremia. The performance and utility of this test remains to be established, but its sensitivity and specificity have been found to be around 95% and 100% respectively, when performed on blood cultures (Sepúlveda et al., 1990; Cobos-Triguero et al., 2017; Parmeland et al., 2021). Neither the *β*-Lacta test nor the oxidase test require additional sample preparation steps, consumables, or specialist training. The results of the present study suggest they can be easily integrated into laboratory workflows.

### Performance and feasibility

The diagnostic performance of the ePlex blood culture identification panel was poorer than previously reported (Schmitz and Tang, 2018; Huang et al., 2019; Bryant et al., 2020; Carroll et al., 2020; Krifors et al., 2020; Oberhettinger et al., 2020), with a sensitivity of just 78% for antibiotic resistances and a very low specificity and negative predictive value for pathogen identification. The resistance identification results should be interpreted with caution since the patients in this study mostly had community-acquired bacteremia with a relatively low prevalence of antibiotic resistance. It is noteworthy however that there were two false negative results with the ePlex assay for methicillin-resistant staphylococci, which could have had serious clinical consequences. The low specificity in pathogen identification is mainly due to the poor performance of the panGram gene search, which was implicated in 89% of false positive results. This panGram gene search was not systematically included in the diagnostic performance analyses in previous studies. Finally, the low sensitivity of this approach in pathogen identification is related to a few cases of bacteremia with opportunistic but not particularly virulent pathogens not included in the ePlex panel (**Table 4**).

**Table 4:** List of discrepancies between ePlex blood culture identification panel and culture methods.

	Details	n
**Pathogen not in ePlex panel (n = 8)**	*Granulicatella adiacens*	1
	*Methylobacterium mesophilicum*	1
	*Sphingomonas paucimobilis*	1
	*Prevotella melaninogenica*	1
	*Moraxella*	1
	*Parvimonas micra*	1
	*Alcaligenes xylosoxidans*	1
	*Clostridium paraputrificum*	1
**Pathogen in panel but not detected (n=5)**	*Fusobacterium necrophorum*	1
	*Pseudomonas aeruginosa*	1
	*Staphylococcus aureus*	1
	*Coagulase negative staphylococcus*	1
	*Enterococcus faecalis*	1
**Resistance not in ePlex panel (n=2)**	*Hypercephalosporinase (Pseudomonas A.)* *Ofloxacine resistance*	1 1
**Resistance in panel but not detected (n=2)**	*MecA (S. aureus and S. hominis)*	2

ePlex assay results differed from conventional culture results in 12 pathogens (8 off-panel microorganisms and 5 negative PCR results for a microorganism in the panel) and for 4 forms of antibiotic resistance [the MecA gene, included in the panel (false negatives), in two cases and hypercephalosporinase resistance in a *Pseudomonas aeruginosa* and ofloxacin resistance in *Escherichia coli* (true negatives)].

In contrast, the diagnostic performance of the three rapid tests was excellent, with sensitivities and specificities of 100%. While the performance and clinical benefit of the GeneXpert MRSA/SA PCR test is well established, this is not the case for the *β*-Lacta and oxidase tests (Parta et al., 2009; Brown and Paladino, 2010; Davies et al., 2012). The *β*-Lacta test is known to be less sensitive to enterobacteria resistant to third-generation cephalosporins by AmpC overproduction, which hydrolyzes the probe enzyme, HMRZ-86, less efficiently than extended-spectrum *β*-lactamases and carbapenemases do (Renvoise et al., 2013; Morosini et al., 2014). It should be noted that we reported none hypercephalosporinase in the sample, which could contribute to the very good diagnostic performances we obtained in the study. In terms of implementation, these rapid tests were found to be easy-to-use and did not slow down the biochemical testing workflow for positive blood cultures. The time from positive blood culture to rapid test results was equal or shorter than the time from positive blood culture to Gram stain results in the reference period. Surprisingly the multitest test protocol was 1 h faster on average than the ePlex assays. This may be because laboratory technicians interpreted Gram stain results sooner when they knew other tests depended on them.

### Therapeutic implication

One of the objectives of this study was to determine whether rapid tests could reduce the time to appropriate antibiotic therapy, avoiding the wait for conventional culture results, which are only provided during working hours in the two hospitals in the study. The cases in this study included both community and hospital-acquired bacteremia with typical rates of inadequate empirical antibiotic therapy (23%) and in-hospital mortality (11%) (Robineau et al., 2018). Our results support the use of rapid techniques for blood culture testing, since they were associated with shorter delays from blood collection to appropriate antibiotic therapy and a greater likelihood of appropriate antibiotic therapy within 24 h of blood sampling (Caliendo et al., 2013; Banerjee et al., 2015; Timbrook et al., 2017; Miller et al., 2018; Oberhettinger et al., 2020). None of these associations were statistically significant, but the effect of the rapid tests is masked somewhat by the time between blood sampling and positive blood culture results having been 2–3 h shorter in the reference period than in the m-PCR and multitest periods.

The use of rapid techniques was not associated with reduced morbidity or mortality, possibly because the study was underpowered to detect this. Another limitation of the study may be that the severity of patients’ symptoms differed, but not significantly, between the three periods. This could explain why no significant difference was observed in the consumption of broad-spectrum antibiotics.

## Conclusion

In these patients with positive blood cultures, the diagnostic performance of multiple rapid tests performed according to a decision algorithm was excellent and superior to that of ePlex assay m-PCR tests. Both rapid techniques were easily incorporated into the laboratory workflow alongside conventional cultures and led to patients receiving appropriate antibiotic therapy sooner. Larger studies with a greater prevalence of resistant pathogens are required to estimate the impact of these tests on length of hospital stay and mortality.

## Data availability statement

The raw data supporting the conclusions of this article will be made available by the authors, without undue reservation.

## Ethics statement

The studies involving human participants were reviewed and approved by Declaration of Helsinki. Written consent was not required because of the observational nature of the study. All patients received general information about research activities and data management policies. Written informed consent for participation was not required for this study in accordance with the national legislation and the institutional requirements.

## Author contributions

P-AC, EV, NR, LP, and CP designed the study. P-AC and NR organized and performed the data collection. P-AC and EV performed the statistical analysis. P-AC, EV, NR, LP, and CP analyzed and interpreted the data. P-AC and EV wrote the manuscript. All authors had full access to the study data, contributed to drafting the manuscript or critically revised its content, approved the final version of the manuscript, and take responsibility for the integrity of the data and the accuracy of the data analysis. EV is the guarantor of the paper. All authors contributed to the article and approved the submitted version.

## References

[B1] Banerjee R. Teng C. B. Cunningham S. A. Ihde S. M. Steckelberg J. M. Moriarty J. P.. (2015). Randomized trial of rapid multiplex polymerase chain reaction–based blood culture identification and susceptibility testing. Clin. Infect. Dis. 61 (7), 1071–1080. doi: 10.1093/cid/civ447 26197846 PMC4560903

[B2] Barlam T. F. Cosgrove S. E. Abbo L. M. MacDougall C. Schuetz A. N. Septimus E. J.. (2016). Executive summary: implementing an antibiotic stewardship program: guidelines by the infectious diseases society of America and the society for healthcare epidemiology of America. Clin. Infect. Dis. 62 (10), 1197–1202. doi: 10.1093/cid/ciw217 27118828

[B3] Brown J. Paladino J. A. (2010). Impact of rapid methicillin-resistant staphylococcus aureus polymerase chain reaction testing on mortality and cost effectiveness in hospitalized patients with bacteraemia: a decision model. PharmacoEconomics. 28 (7), 567–575. doi: 10.2165/11533020-000000000-00000 20550222

[B4] Bryant S. Almahmoud I. Pierre I. Bardet J. Touati S. Maubon D.. (2020). Evaluation of microbiological performance and the potential clinical impact of the ePlex® blood culture identification panels for the rapid diagnosis of bacteremia and fungemia. Front. Cell Infect. Microbiol. 10, 594951. doi: 10.3389/fcimb.2020.594951 33324578 PMC7726344

[B5] Caliendo A. M. Gilbert D. N. Ginocchio C. C. Hanson K. E. May L. Quinn T. C.. (2013). Better tests, better care: improved diagnostics for infectious diseases. Clin. Infect. Dis. 57 (suppl 3), S139–S170. doi: 10.1093/cid/cit578 24200831 PMC3820169

[B6] Carroll K. C. Reid J. L. Thornberg A. Whitfield N. N. Trainor D. Lewis S.. (2020). Clinical performance of the novel GenMark dx ePlex blood culture ID gram-positive panel. J. Clin. Microbiol. 58 (4), e01730–e01719. doi: 10.1128/JCM.01730-19 31996444 PMC7098771

[B7] CASFM/EUCAST (2019) CASFM/EUCAST V1.1. Available at: https://www.sfm-microbiologie.org/2019/01/07/casfm-eucast-2019.

[B8] Cassini A. Högberg L. D. Plachouras D. Quattrocchi A. Hoxha A. Simonsen G. S.. (2019). Attributable deaths and disability-adjusted life-years caused by infections with antibiotic-resistant bacteria in the EU and the European economic area in 2015: a population-level modelling analysis. Lancet Infect. Dis. 19 (1), 56–66. doi: 10.1016/S1473-3099(18)30605-4 30409683 PMC6300481

[B9] Cobos-Triguero N. Zboromyrska Y. Morata L. Alejo I. de la Calle C. Vergara A.. (2017). Time-to-positivity, type of culture media and oxidase test performed on positive blood culture vials to predict pseudomonas aeruginosa in patients with gram-negative bacilli bacteraemia. Rev. Espanola Quimioter Publicacion Of Soc. Espanola Quimioter. 30 (1), 9–13. 27897434

[B10] Compain F. Bensekhri H. Rostane H. Mainardi J. L. Lavollay M. (2015). β LACTA test for rapid detection of enterobacteriaceae resistant to third-generation cephalosporins from positive blood cultures using briefly incubated solid medium cultures. J. Med. Microbiol. 64 (10), 1256–1259. doi: 10.1099/jmm.0.000157 26307077

[B11] Davies J. Gordon C. L. Tong S. Y. C. Baird R. W. Davis J. S. (2012). Impact of results of a rapid staphylococcus aureus diagnostic test on prescribing of antibiotics for patients with clustered gram-positive cocci in blood cultures. J. Clin. Microbiol. 50 (6), 2056–2058. doi: 10.1128/JCM.06773-11 22493335 PMC3372134

[B12] De Waele J. J. Akova M. Antonelli M. Canton R. Carlet J. De Backer D.. (2018). Antimicrobial resistance and antibiotic stewardship programs in the ICU: insistence and persistence in the fight against resistance. a position statement from ESICM/ESCMID/WAAAR round table on multi-drug resistance. Intensive Care Med. 44 (2), 189–196. doi: 10.1007/s00134-017-5036-1 29288367

[B13] Garnier M. Rozencwajg S. Pham T. Vimont S. Blayau C. Hafiani M.. (2017). Evaluation of early antimicrobial therapy adaptation guided by the BetaLACTA® test: a case-control study. Crit. Care 21 (1), 161. doi: 10.1186/s13054-017-1746-6 28655352 PMC5488410

[B14] Hasso M. Porter V. Simor A. E. (2017). Evaluation of the *β*-lacta test for detection of extended-Spectrum-*β*- lactamase (ESBL)-producing organisms directly from positive blood cultures by use of smudge plates. J. Clin. Microbiol. 55 (12), 3. doi: 10.1128/JCM.01354-17 29021153 PMC5703822

[B15] Huang T. D. Melnik E. Bogaerts P. Evrard S. Glupczynski Y. (2019). Evaluation of the ePlex blood culture identification panels for detection of pathogens in bloodstream infections. J. Clin. Microbiol. 57 (2), e01597–18. doi: 10.1128/JCM.01597-18 30487304 PMC6355516

[B16] Kollef M. H. (2000). Inadequate antimicrobial treatment: an important determinant of outcome for hospitalized patients. Clin. Infect. Dis. 31 (Supplement_4), S131–S138. doi: 10.1086/314079 11017862

[B17] Krifors A. Rådberg G. Golbob S. Omar Z. Svensson C. Heimer D.. (2020). The clinical impact of implementing GenMark ePlex blood culture panels for around-the-clock blood culture identification; a prospective observational study. Infect. Dis. 52 (10), 705–712. doi: 10.1080/23744235.2020.1775882 32522111

[B18] Kumar A. (2010). Early antimicrobial therapy in severe sepsis and septic shock. Curr. Infect. Dis. Rep. 12 (5), 336–344. doi: 10.1007/s11908-010-0128-x 21308515

[B19] Leone M. Bourgoin A. Cambon S. Dubuc M. Albanèse J. Martin C. (2003). Empirical antimicrobial therapy of septic shock patients: adequacy and impact on the outcome*. Crit. Care Med. 31 (2), 462–467. doi: 10.1097/01.CCM.0000050298.59549.4A 12576952

[B20] Liesenfeld O. Lehman L. Hunfeld K. P. Kost G. (2014). Molecular diagnosis of sepsis: new aspects and recent developments. Eur. J. Microbiol. Immunol. 4 (1), 1–25. doi: 10.1556/EuJMI.4.2014.1.1 PMC3955828 24678402

[B21] Miller J. M. Binnicker M. J. Campbell S. Carroll K. C. Chapin K. C. Gilligan P. H.. (2018). A guide to utilization of the microbiology laboratory for diagnosis of infectious diseases: 2018 update by the infectious diseases society of America and the American society for microbiologya. Clin. Infect. Dis. 67 (6), e1–94. doi: 10.1093/cid/ciy381 29955859 PMC7108105

[B22] Morosini M. I. Garcia-Castillo M. Tato M. Gijon D. Valverde A. Ruiz-Garbajosa P.. (2014). Rapid detection of -Lactamase-Hydrolyzing extended-spectrum cephalosporins in enterobacteriaceae by use of the new chromogenic lacta test. J. Clin. Microbiol. 52 (5), 1741–1744. doi: 10.1128/JCM.03614-13 24574293 PMC3993668

[B23] Oberhettinger P. Zieger J. Autenrieth I. Marschal M. Peter S. (2020). Evaluation of two rapid molecular test systems to establish an algorithm for fast identification of bacterial pathogens from positive blood cultures. Eur. J. Clin. Microbiol. Infect. Dis. 39 (6), 1147–1157. doi: 10.1007/s10096-020-03828-5 32020397 PMC7225181

[B24] Parmeland L. Bourru T. Kyungu V. Rousseau N. Gleize M. De Beauvoir C.. (2021). A rapid and inexpensive protocol to screen for third generation cephalosporin-resistant and non-fermenting gram-negative rods directly in positive blood cultures. Diagn. Microbiol. Infect. Dis. 101 (2), 115428. doi: 10.1016/j.diagmicrobio.2021.115428 34174522

[B25] Parta M. Goebel M. Matloobi M. Stager C. Musher D. M. (2009). Identification of methicillin-resistant or methicillin-susceptible *Staphylococcus aureus* in blood cultures and wound swabs by GeneXpert. J. Clin. Microbiol. 47 (5), 1609–1610. doi: 10.1128/JCM.00351-09 19261790 PMC2681863

[B26] Renvoise A. Decre D. Amarsy-Guerle R. Huang T. D. Jost C. Podglajen I.. (2013). Evaluation of the lacta test, a rapid test detecting resistance to third-generation cephalosporins in clinical strains of enterobacteriaceae. J. Clin. Microbiol. 51 (12), 4012–4017. doi: 10.1128/JCM.01936-13 24068012 PMC3838025

[B27] Rhodes A. Evans L. E. Alhazzani W. Levy M. M. Antonelli M. Ferrer R.. (2017). Surviving sepsis campaign: international guidelines for management of sepsis and septic shock: 2016. Intensive Care Med. 43 (3), 304–377. doi: 10.1007/s00134-017-4683-6 28101605

[B28] Rivers E. Nguyen B. Havstad S. Ressler J. Muzzin A. Knoblich B.. (2001). Early goal-directed therapy in the treatment of severe sepsis and septic shock. N Engl. J. Med. 345 (19), 1368–1377. doi: 10.1056/NEJMoa010307 11794169

[B29] Robineau O. Robert J. Rabaud C. Bedos J. P. Varon E. Péan Y.. (2018). Management and outcome of bloodstream infections: a prospective survey in 121 French hospitals (SPA-BACT survey). Infect. Drug Resist. 11, 1359–1368. doi: 10.2147/IDR.S165877 30214256 PMC6124465

[B30] Rodrigues C. Siciliano R. F. Filho H. C. Charbel C. E. de Carvalho Sarahyba da Silva L. Baiardo Redaelli M.. (2019). The effect of a rapid molecular blood test on the use of antibiotics for nosocomial sepsis: a randomized clinical trial. J. Intensive Care 7 (1), 37. doi: 10.1186/s40560-019-0391-3 31367384 PMC6647273

[B31] Salimnia H. Fairfax M. R. Lephart P. R. Schreckenberger P. DesJarlais S. M. Johnson J. K.. (2016). Evaluation of the FilmArray blood culture identification panel: results of a multicenter controlled trial. J. Clin. Microbiol. 54 (3), 687–698. doi: 10.1128/JCM.01679-15 26739158 PMC4767991

[B32] Schmitz J. E. Tang Y. W. (2018). The GenMark ePlex ^®^: another weapon in the syndromic arsenal for infection diagnosis. Future Microbiol. 13 (16), 1697–1708. doi: 10.2217/fmb-2018-0258 30547684 PMC6439521

[B33] Seifert H. (2009). The clinical importance of microbiological findings in the diagnosis and management of bloodstream infections. Clin. Infect. Dis. 48 (s4), S238–S245. doi: 10.1086/598188 19374579

[B34] Sepúlveda J. L. Stager C. E. Davis J. R. (1990). Rapid presumptive identification of gram-negative rods directly from blood cultures by simple enzymatic tests. J. Clin. Microbiol. 28 (2), 177–181. doi: 10.1128/jcm.28.2.177-181.1990 2107196 PMC269571

[B35] Southern T. R. VanSchooneveld T. C. Bannister D. L. Brown T. L. Crismon A. S. Buss S. N.. (2015). Implementation and performance of the BioFire FilmArray® blood culture identification panel with antimicrobial treatment recommendations for bloodstream infections at a midwestern academic tertiary hospital. Diagn. Microbiol. Infect. Dis. 81 (2), 96–101. doi: 10.1016/j.diagmicrobio.2014.11.004 25488272

[B36] Timbrook T. T. Morton J. B. McConeghy K. W. Caffrey A. R. Mylonakis E. LaPlante K. L. (2017). The effect of molecular rapid diagnostic testing on clinical outcomes in bloodstream infections: a systematic review and meta-analysis. Clin. Infect. Dis. 64 (1), 15–23. doi: 10.1093/cid/ciw649 27678085

[B37] Vardakas K. Z. Anifantaki F. I. Trigkidis K. K. Falagas M. E. (2015). Rapid molecular diagnostic tests in patients with bacteremia: evaluation of their impact on decision making and clinical outcomes. Eur. J. Clin. Microbiol. Infect. Dis. 34 (11), 2149–2160. doi: 10.1007/s10096-015-2466-y 26329038

[B38] Walker T. Dumadag S. Lee C. J. Lee S. H. Bender J. M. Cupo Abbott J.. (2016). Clinical impact of laboratory implementation of verigene BC-GN microarray-based assay for detection of gram-negative bacteria in positive blood cultures. J. Clin. Microbiol. 54 (7), 1789–1796. doi: 10.1128/JCM.00376-16 27098961 PMC4922078

[B39] Yokota P. K. O. Marra A. R. Martino M. D. V. Victor E. S. Durão M. S. Edmond M. B.. (2014). Impact of appropriate antimicrobial therapy for patients with severe sepsis and septic shock – a quality improvement study. PloS One 9 (11), e104475. doi: 10.1371/journal.pone.0104475 25375775 PMC4222820

