# Supplementary material for: Shortening identification times: comparative observational study of three early blood culture testing protocols
Source: Front Cell Infect Microbiol. 2023 Jul 18;13:1192002. doi: 10.3389/fcimb.2023.1192002 (PMC10390722; doi:10.3389/fcimb.2023.1192002)
Supplement: Supplementary file 1 [file DataSheet_1.docx]

**SUPPLEMENTAL MATERIAL**

**MATERIALS AND METHODS**

*Exclusion criteria*

Duplicate results were excluded. Isolated positive blood culture bottles (i.e. no other positive bottles for the patient) with gram-positive cocci in clusters were considered contaminated and excluded. For patients with several positive blood cultures, the first one was retained, unless this was positive for gram-positive cocci in clusters, in which case the second positive bottle (from the same or a different pair of bottles) was considered.

*Antimicrobial susceptibility testing*

Antimicrobial susceptibility tests and minimal inhibitory concentrations were interpreted according to European Committee on Antimicrobial Susceptibility Testing clinical breakpoints (EUCAST 2018). Extended-spectrum β-lactamases were detected using double-disc synergy tests.

*Multiplex PCR*

Multiplex PCR tests with the ePlex blood culture identification panel were performed on positive blood culture bottles with the appropriate cartridge (gram-positive, gram-negative or fungal), as determined by the Gram stain results. All three ePlex cartridges contain a pan-gram-positive pan-gram-negative, or pan-Candida probe as quality control should the Gram stain result have been misread (**Table 1**). If the quality-control probe was positive, the same blood culture was tested again using the corresponding cartridge. The blood culture samples were loaded directly into the port of the respective cartridge without manual preprocessing. Extraction, amplification and detection were all performed automatically in the cartridge, with a total analysis time of less than 2 h. Results (laboratory reports) were made available on the hospital computer system, including the causative pathogen(s) and possible antibiotic resistances, and recommendations for (starting or modifying) antibiotic therapy ***(Supplemental Table 1).***

*Rapid tests*

Three types of rapid tests were used in combination based on Gram stain results: *β*-Lacta and oxidase tests for gram-negative bacteria and an MRSA/SA PCR test for gram-positive cocci in clusters.

The Bactident oxidase test (Merck, Darmstadt, Germany) is a colorimetric phenotypic test to identify the presence of cytochrome oxidase-producing species (Pseudomonas, Aeromonas, Alcaligenes, Branhamella, Bordetella, Moraxella, Neisseria, and Vibrio species, and certain strains of Brucella, Haemophilus and Pasteurella). It can be used in clinical practice (as it was here) to distinguish (oxidase-positive) Pseudomonas aeruginosa infections from enterobacterial and other non-fermenting (oxidase-negative) gram-negative bacteremia. Cytochrome oxidase oxidizes the phenylenediamine reagent (colorless or pale yellow) in the presence of atmospheric oxygen and cytochrome C, into indophenol, a purple to violet compound.

The β-Lacta test (Bio-Rad®, Marnes la Coquette, France) is a rapid colorimetric diagnostic test to identify enterobacterial strains resistant to broad-spectrum cephalosporins, based on the cleavage of a chromogenic cephalosporin, HMRZ-86. The test detects any enzymatic activity that could lead to the hydrolysis of broad-spectrum cephalosporins, without distinguishing between the mechanisms of extended-spectrum β-lactamases, cephalosporinase, or type B and D carbapenemases.

For the study, samples (10 mL) of positive culture broth (aerobic or anaerobic) were divided equally into two dry tubes with separating gel (Greiner Bio-One, Courtaboeuf, France) and centrifuged for 5 min at 2800 g. After removal of the supernatant, the bacterial pellet on the surface of the separator gel of the first tube was scratched with an inoculation loop and smeared on an oxidase test strip. The test was considered positive if the smeared part of the strip turned purple, whereas delayed reactions or an absence of coloring were considered negative tests.

If the oxidase test was negative, a BetaLacta Test (Bio-Rad®) was performed on the bacterial pellet from the second tube. This was suspended in a tube containing 1 drop of reagents 1 and 2 from the kit. After 10 min, a first reading was performed at room temperature. A reaction was considered positive if the yellow indicator turned orange/red. If the first reading was negative, a second reading was performed after 10 min incubation at 30°C. Tests were considered negative when the sample did not change color (1–3).

The diagnostic accuracy of a rapid two-test protocol involving oxidase and β-Lacta tests has been evaluated previously on 294 positive blood cultures (4).

The GeneXpert MRSA/SA test (Cepheid, Sunnyvale, CA) is a real-time PCR test, designed to identify methicillin-resistant *Staphylococcus aureus*. It contains several probes that target three nucleotide sequences: the *spa* gene encoding *S. aureus* protein A, the *mecA* gene encoding the methicillin-resistance of the PLP2a protein, and genes from the staphylococcal cassette chromosome *mec* to detect methicillin-resistance genes other than *mecA*. The PCR tests were performed directly on positive blood culture samples and results were available within 2 h.

*Power calculations*

Estimates of the required sample size were obtained based on estimated times from blood culture positivity to rapid technique results of 3 h for the m-PCR and multitest periods and 4 h for the reference period. The corresponding sample size required to detect a difference of 1 h in this delay with an α-level of 0.05 and a power (1 − β) of 0.9 was 85 patients in each of the three periods. We decided to include 90 patients in each period.

*Data protection*

All patients were provided with general information about research activities and data management policies in keeping with French data protection law. No data other than those directly related to the study were collected.

***Supplemental table 1: Antibiotic treatment guidelines based on ePlex multiplex PCR results***

| **Pathogen** | **Resistance** | **First line antibiotic therapy** | **Second line Antibiotic therapy** | **β-lactamase allergy** |
| --- | --- | --- | --- | --- |
| ***Salmonella,***  ***P. mirabilis,***  ***E. coli,***  ***K. pneumoniae*** | - | Cefotaxime  +/- Amikacin if shock | Cefoxitin, Ceftriaxone, Cefepim, Ceftazidim, Imipenem, Meropenem | Aztreonam + amikacin |
|  | CTX-M | Imipenem, Meropenem  +/- Amikacin if shock | Cefoxitin, Temocillin, Ceftolozan-tazobactam, Ceftazidim-avibactam | Aztreonam + amikacin |
|  | VIM, KPC, OXA, IMP, NDM | Ceftazidim-avibactam  +/- Amikacin if shock | Colistin | Aztreonam + amikacin |
| ***K. oxytoca*** | - | Cefepim  +/- Amikacin if shock | Imipenem, Meropenem | Aztreonam + amikacin |
|  | CTX-M | Imipenem, Meropenem  +/- Amikacin if shock | Ceftolozan-tazobactam, Ceftazidim-avibactam | Aztreonam + amikacin |
|  | VIM, KPC, OXA, IMP, NDM | Ceftazidim-avibactam  +/- Amikacin if shock | Colistin | Colistin |
| ***E. non cloacae complex,***  ***S. marcescens*** | - | Cefepim  +/- Amikacin if shock | Imipenem, Meropenem | Aztreonam + amikacin |
|  | CTX-M | Imipenem, Meropenem  +/- Amikacin if shock | Ceftolozan-tazobactam, Ceftazidim-avibactam | Aztreonam + amikacin |
|  | VIM, KPC, OXA, IMP, NDM | Ceftazidim-avibactam, Ceftolozan-tazobactam  + Amikacin |  | Aztreonam + amikacin |
| ***E. cloacae complex*** | - | Cefepim  +/- Amikacin if shock | Imipenem, Meropenem | Aztreonam + amikacin |
|  | CTX-M | Imipenem, Meropenem  +/- Amikacin if shock | Ceftolozan-tazobactam, Ceftazidim-avibactam | Amikacin |
|  | VIM, KPC, OXA, IMP, NDM | Ceftazidim-avibactam  + Amikacin / Colistin |  |  |
| ***Citrobacter,***  ***Other Proteus, M. morganii, Serratia non marcescens*** | - | Cefepim  +/- Amikacin if shock | Imipenem, Meropenem, Ceftolozan-tazobactam, Ceftazidim-avibactam | Aztreonam + amikacin |
|  | CTX-M | Imipenem, Meropenem  + Amikacin | Ceftolozan-tazobactam, Ceftazidim-avibactam | Aztreonam + Amikacin |
|  | VIM, KPC, OXA, IMP, NDM | Ceftazidim-avibactam  + Amikacin / Colistin |  |  |
| ***P. aeruginosa*** | - | Ceftazidim, Piperacillin-tazobactam  +/- Amikacin if shock | Imipenem, Meropenem | Aztreonam + Amikacin |
|  | CTX-M | Imipenem, Meropenem  +/- Amikacin if shock | Ceftolozan-tazobactam, Ceftazidim-avibactam | Ciprofloxacin + Amikacin |
|  | VIM, KPC, OXA, IMP, NDM | Aztreonam + Amikacin | Ceftolozan-tazobactam, Ceftazidim-avibactam  + Amikacin | Aztreonam + Amikacin |
| ***S. maltophilia*** | - | Ticarcillin-Clavulanate, Trimethoprim-sulfamethoxazole  + Amikacin | Ceftolozan-tazobactam, Ceftazidim-avibactam | Trimethoprim-sulfamethoxazole + Amikacin |
|  | CTX-M | Trimethoprim-sulfamethoxazole  + Amikacin | Ceftolozan-tazobactam, Ceftazidim-avibactam | Trimethoprim-sulfamethoxazole + Amikacin |
|  | VIM, KPC, OXA, IMP, NDM | Trimethoprim-sulfamethoxazole  + Amikacin | Colistin | Trimethoprim-sulfamethoxazole + Amikacin |
| ***A. baumanii*** | - | Imipenem, Meropenem  +/- Amikacin if shock |  | Amikacin |
|  | CTX-M | Imipenem, Meropenem  + Amikacin if shock |  | Amikacin |
|  | VIM, KPC, OXA, IMP, NDM | Colistin |  |  |
| ***H. influenzae*** | - | Amoxicillin-clavulanate, cefotaxime | Piperacillin-tazobactam, Cefazolin, Cefoxitin, Ceftriaxone, Cefepim, Ceftazidim | Levofloxacin + Amikacin |
| ***N. meningitis*** | - | Cefotaxime, Ceftriaxone  +/- Gentamycin if shock |  | Levofloxacin + Gentamycin |
| ***B. fragilis*** | - | + Metronidazole | Amoxicillin-clavulanate, Piperacillin-tazobactam, imipenem, meropenem | Metronidazole |
| ***F. necrophorum*** | - | + Metronidazole | Amoxicillin-clavulanate, Cefotaxime, Ceftriaxone, Ceftazidim | Metronidazole |
| ***S. aureus*** | - | Cloxacillin, cefazolin  +/- Gentamycin if shock | Daptomycin (except pulmonary infection), Ceftaroline, Imipenem, Meropenem | Daptomycin |
|  | *mecA*, *mecC* | Daptomycin (except pulmonary infection)  +/- Gentamycin if shock | Vancomycin, Ceftaroline |  |
| ***Coagulase negative staphylococcus**** | - | Cloxacillin, cefazolin  +/- Gentamycin if shock | Daptomycin (except pulmonary infection), Vancomycin, Imipenem, Meropenem | Daptomycin |
|  | *mecA*, *mecC* | Vancomycin | Daptomycin |  |
| ***S. pneumoniae*** | - | Amoxicillin, Cefotaxime  +/- Gentamycin if shock | Cefazolin, Cefoxitin, Ceftazidim, Cefepim, Ceftriaxone | Daptomycin (except pulmonary infection), Levofloxacin |
| ***S. pyogenes*** | - | Amoxicillin + Clindamycin  +/- Gentamycin if shock | Clindamycin +  Amoxicillin-Clavulanate, Cefazolin, Cefoxitin, Cefotaxime, Ceftriaxone, Ceftazidim, Cefepim, Piperacillin-tazobactam, Imipenem, Meropenem, Daptomycin | Daptomycin + Clindamycin |
| ***Other Streptococcus*** | - | Amoxicillin  +/- Gentamycin if shock | Amoxicillin-Clavulanate, Cefazolin, Cefoxitin, Cefotaxime, Ceftriaxone, Ceftazidim, Cefepim, Piperacillin-tazobactam, Imipenem, Meropenem, Daptomycin | Daptomycin |
| ***E. faecalis*** | - | Amoxicillin  +/- Gentamycin if shock | Amoxicillin-Clavulanate, Piperacillin, imipenem, Daptomycin, vancomycin | Daptomycin + gentamycin |
|  | van A, van B | Amoxicillin  +/- Gentamycin if shock | Amoxicillin-Clavulanate, Piperacillin, imipenem, Daptomycin | Daptomycin + gentamycin |
| ***E. faecium*** | - | Vancomycin  +/- Gentamycin if shock | Daptomycin |  |
|  | van A, van B | Vancomycin  +/- Gentamycin if shock |  |  |
| ***Other Enterococcus*** | - | Amoxicillin + Gentamycin | Amoxicillin-clavulanate, Piperacillin, Daptomycin | Daptomycin + Gentamycin |
|  | van A, van B | Amoxicillin + Gentamycin | Amoxicillin-clavulanate, Piperacillin, Daptomycin | Daptomycin + Gentamycin |
| ***Listeria*** | - | Amoxicillin, Trimethoprim-sulfamethoxazole  +/- Gentamycin if shock | Imipenem, Meropenem, Vancomycin | Trimethoprim-sulfamethoxazole  +/- Gentamycin if shock |
| ***Cutibacterium***** | - | Amoxicillin | Ofloxacin | Ofloxacin |
| ***Coryne-bacterium***** | - | Amoxicillin |  |  |
| ***Bacillus spp***** | - | Ofloxacin  +/- Gentamycin if shock | Vancomycin |  |
| ***Candida spp*** |  | Caspofungin |  |  |
| ***Cryptococcus*** |  | Liposomal Amphotericin B | Fluconazole |  |

* *Gram-positive cocci in clusters: treat after testing two more pairs of blood cultures if infection probable (signs of sepsis or vascular/cardiac involvement and > 1 pair of positive blood cultures); sample two more blood cultures and do not treat if probable contamination (no sign of sepsis and no intravascular/cardiac material and only one pair of positive blood cultures).*

** Opportunistic pathogen: *treat after testing one more pair of blood cultures if infection probable (signs of sepsis or vascular/cardiac involvement and > 1 pair of positive blood cultures); sample one more pair of BC and don’t treat if contamination probable (no sign of sepsis or vascular/cardiac involvement and only one positive blood culture bottle*

***Supplemental table 2: Antibiotic treatment guidelines based on Gram stain and/or rapid test results***

| Gram Stain | Supplemental information | Fist line antibiotic therapy | Antibiotic therapy if β-lactamase allergy |
| --- | --- | --- | --- |
| *GPCC** | Negative Xpert MRSA/SA | Cloxacillin or cefazolin  +/- Gentamycin if shock | Daptomycin  +/- Gentamycin if septic shock |
|  | Positive Xpert MRSA/SA or nosocomial BSI and quick test unavailable | Daptomycin  +/- Gentamycin if shock |  |
| *GP cocci in chains* |  | Amoxicillin  +/- Gentamycin if shock | Daptomycin  +/- Gentamycin if septic shock |
| *Gram-negative bacilli* | Community BSI or  negative β-Lacta test and negative oxidase test if available | Cefotaxime  +/- Metronidazole if anaerobic GNB suspected  +/- Amikacin if shock | Aztreonam + Amikacin  +/- Metronidazole if anaerobic GNB suspected |
|  | Nosocomial BSI and quick test unavailable | Piperacillin-tazobactam  +/- Amikacin if shock | Aztreonam + Metronidazole  +/- Amikacin if septic shock |
|  | Positive oxidase test if available | Piperacillin-tazobactam or Ceftazidim  +/- Amikacin if shock | Ciprofloxacin + Amikacin |
|  | Positive β-Lacta test and negative oxidase test if available | Imipenem or Meropenem  +/- Amikacin if shock | Ciprofloxacin + Amikacin |

* *Gram-positive cocci in clusters: treat after testing two more pairs of blood cultures if infection probable (signs of sepsis or vascular/cardiac involvement and > 1 pair of positive blood cultures); sample two more blood cultures and do not treat if probable contamination (no sign of sepsis and no intravascular/cardiac material and only one pair of positive blood cultures*

|  | **ePlex BCI panel**  **(n = 90)** | **Xpert MRSA/SA**  **(n = 30)** | **β-Lacta Test**  **(n = 38)** | **Oxidase Test**  **(n = 38)** |
| --- | --- | --- | --- | --- |
| **Pathogen identification or orientation** |  |  |  |  |
| Sensitivity | 93 | - | - | 100 |
| Specificity | 40 | - | - | 100 |
| Positive predictive value | 88 | - | - | 100 |
| Negative predictive value | 54 | - | - | 100 |
| **Bacterial resistance identification** |  |  |  |  |
| Sensitivity | 78 | 100 | 100 | - |
| Specificity | 100 | 100 | 100 | - |
| Positive predictive value | 100 | 100 | 100 | - |
| Negative predictive value | 98 | 100 | 100 | - |

***Supplemental table 3: Diagnostic performance of blood culture tests***

*Data are reported as percentages.*

*BCI, blood culture identification*

*Discrepancies appeared with ePlex assay were: (i) missing 2 mecA genes, (ii) misidentifying 8 pangram and one Staphylococcus hominis.*

|  | m-PCR period  (n= 90) | Reference period  (n= 90) | Multitest period  (n= 90) | *p* |
| --- | --- | --- | --- | --- |
| Carbapenems  Patients, *n*  Duration if using, *days* | 6 (7)  10 (10-10.8) | 3 (3)  7 (6-7.5) | 9 (9)  2 (1-7) | 0,199  0.163 |
| Piperacillin-tazobactam  Patients, *n*  Duration if using, *days* | 18 (20)  5 (2.25-10) | 26 (29)  4 (2-8.75) | 17 (19)  6 (3-10) | 0,204  0.714 |
| C4G  Patients, *n*  Duration if using, *days* | 7 (8)  4 (2.5-8.5) | 3 (3)  3 (2-5) | 4 (5)  4.5 (1.8-7.3) | 0,387  0.811 |
| Parenteral C3G  Patients, *n*  Duration if using, *days* | 56 (62)  5.5 (2-10.3) | 56 (62)  5.5 (2-10) | 51 (58)  3 (1-6.5) | 0,798  0.033 |
| Aminoglycosides  Patients, *n*  Duration if using, *days* | 19 (21)  1 (1-2) | 15 (17)  1 (1-1.5) | 24 (27)  1 (1-2.3) | 0,258  0.544 |
| Vancomycin  Patients, *n*  Duration if using, *days* | 5 (6)  4 (1-4) | 9 (10)  4 (3-13) | 13 (15)  5 (1-13) | 0,131  0,501 |
| Daptomycin  Patients, *n*  Duration if using, *days* | 7 (8)  4 (2.5-5) | 7 (8)  7 (3.5-11.5) | 11 (12)  5 (2-14) | 0.485  0.473 |
| Linezolid  Patients, *n*  Duration if using, *days* | 0 | 0 | 0 |  |

***Supplemental table 4: Consumption of broad-spectrum antibiotics by study period***

**REFERENCES**

1. Renvoise A, Decre D, Amarsy-Guerle R, Huang TD, Jost C, Podglajen I, et al. Evaluation of the Lacta Test, a Rapid Test Detecting Resistance to Third-Generation Cephalosporins in Clinical Strains of Enterobacteriaceae. J Clin Microbiol. 2013 Dec 1;51(12):4012–7.

2. Compain F, Bensekhri H, Rostane H, Mainardi JL, Lavollay M. β LACTA test for rapid detection of Enterobacteriaceae resistant to third-generation cephalosporins from positive blood cultures using briefly incubated solid medium cultures. J Med Microbiol. 2015 Oct 1;64(10):1256–9.

3. Hasso M, Porter V, Simor AE. Evaluation of the ␤-Lacta Test for Detection of Extended-Spectrum-␤- Lactamase (ESBL)-Producing Organisms Directly from Positive Blood Cultures by Use of Smudge Plates. J Clin Microbiol. 2017;55(12):3.

4. Parmeland L, Bourru T, Kyungu V, Rousseau N, Gleize M, De Beauvoir C, et al. A rapid and inexpensive protocol to screen for third generation cephalosporin-resistant and non-fermenting Gram-negative rods directly in positive blood cultures. Diagn Microbiol Infect Dis. 2021 Oct;101(2):115428.
